# Solitary recurrence of prostate cancer surrounded by seminal vesicle/vas deferens‐like epithelium

**DOI:** 10.1002/iju5.12168

**Published:** 2020-07-30

**Authors:** Hajime Takamori, Tomomi Kamba, Shinji Sumiyoshi, Toyonori Tsuzuki, Soki Kashima, Takayuki Yoshino, Takeshi Sano, Takayuki Goto, Atsuro Sawada, Shusuke Akamatsu, Takashi Kobayashi, Toshinari Yamasaki, Takashi Mizowaki, Osamu Ogawa, Takahiro Inoue

**Affiliations:** ^1^ Department of Urology Kyoto University Hospital Kyoto Japan; ^2^ Department of Urology Kumamoto University Hospital Kumamoto Japan; ^3^ Department of Diagnostic Pathology Kyoto University Hospital Kyoto Japan; ^4^ Department of Surgical Pathology Aichi Medical University Hospital Nagakute Japan; ^5^ Department of Radiation Oncology and Image‐Applied Therapy Kyoto University Hospital Kyoto Japan; ^6^ Department of Nephro‐urologic Surgery Mie University Hospital Tsu Japan

**Keywords:** metastasis‐directed therapy, oligorecurrence, prostate cancer, seminal vesicle, vas deferens

## Abstract

**Introduction:**

Clinical recurrence of prostate cancer after curative treatment with a limited number of metastases is often termed as oligorecurrence. We report a case of solitary recurrence of prostate cancer surrounded by epithelium of the seminal vesicle or vas deferens.

**Case presentation:**

A 54‐year‐old man diagnosed with localized prostate cancer underwent radiation therapy. Six years later, imaging studies detected a solitary recurrence. We performed metastasectomy, and histopathological examination revealed the metastatic lesion surrounded by the epithelium of the seminal vesicle or vas deferens. Surgical resection achieved a complete biochemical response.

**Conclusion:**

We presented with a case of prostate cancer metastasis surrounded by the epithelium of the seminal vesicle or vas deferens.

**Abbreviations & Acronyms**
ADT: androgen deprivation therapy
AR: androgen receptor
CRPC: castration‐resistant prostate cancer
CT: computed tomography
IMRT: intensity‐modulated radiation therapy
MDT: metastasis‐directed therapy
PCa: prostate cancer
PSA: prostate‐specific antigen
SBRT: stereotactic body radiotherapy

**Keynote message**

We report a rare case of oligorecurrent PCa, in which the metastatic tumor was surrounded by the epithelium of the seminal vesicle or the vas deferens.

## Introduction

Although ADT is the standard treatment option for clinically recurrent PCa after radiotherapy, it is not a curative treatment.^1^ Recently, selected studies have reported that in cases of relapse with three or fewer metastases, often termed oligorecurrences, MDT may delay the initiation of palliative systemic therapy.^2^ We herein report a case of solitary recurrence of PCa after radiotherapy, which achieved a complete biochemical response after surgery. Pathological examination revealed that the metastasis was surrounded by epithelium of the seminal vesicle or vas deferens, which is extremely rare.

## Case presentation

**Fig. 1:**
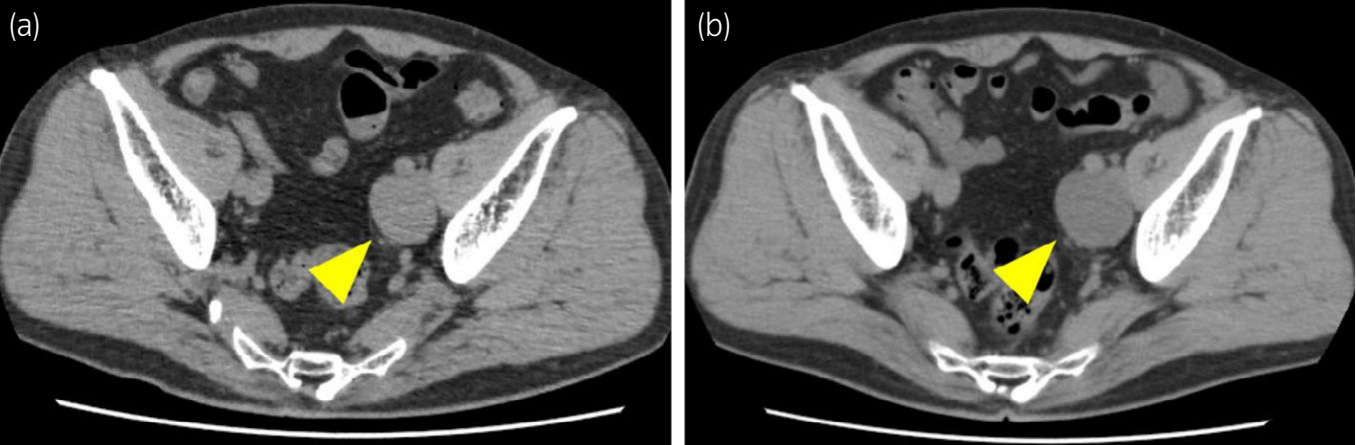
(a) CT scan prior to the initiation of degarelix acetate. Clinical recurrence of PCa. (b) CT scan after the initiation of degarelix acetate, which revealed the enlargement of metastasis.

A 54‐year‐old man presented with gross hematuria. He neither had a history of surgery nor a family history of PCa. He was diagnosed with PCa (PSA level 140 ng/mL, Gleason score 4 + 5, cT3bN0M0). After neoadjuvant 6‐month ADT, a total dose of 78 Gy was administered to the prostate and the seminal vesicles as IMRT.^3^ His PSA level reached nadir at <0.008 ng/mL 3 months after the initiation of IMRT and increased slowly thereafter. Biochemical recurrence occurred 5 years after the IMRT. Imaging studies revealed no metastasis and observation was continued. Six years after the IMRT, a plain CT scan revealed clinical recurrence with a solitary metastatic lesion in the left external iliac area (Fig. 1a). No bone metastasis was detected by bone scintigraphy. We planned external beam radiotherapy to the metastatic lesion after ADT. One month after the initiation of degarelix acetate, the PSA level rapidly elevated from 3.6 to 14.7 ng/mL, even though serum testosterone decreased to a castrate level. CT revealed enlargement of the lesion (Fig. 1b). The patient’s disease status was determined as CRPC with rapid progression. We altered the treatment plan to include the resection of the lesion for histopathologic diagnosis. We performed a laparoscopic metastasectomy.

**Fig. 2:**
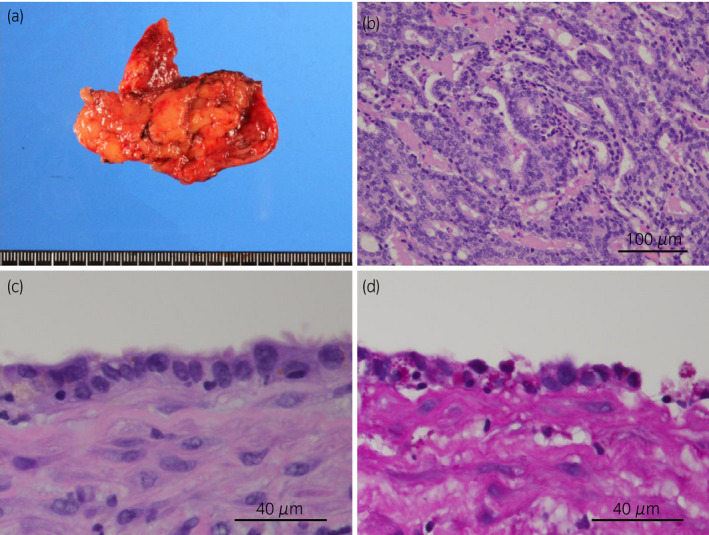
(a) Gross appearance of the resected mass. (b) Histology of the intracystic tissue, demonstrating metastatic adenocarcinoma (hematoxylin and eosin stain). (c) Histology of the cyst wall. The luminal surface was lined with ciliated cuboidal epithelium (hematoxylin and eosin stain). (d) Histology of the cyst wall. The lining epithelium contained lipofuscin in the cytoplasm (Periodic acid‐Schiff reaction).

**Fig. 3:**
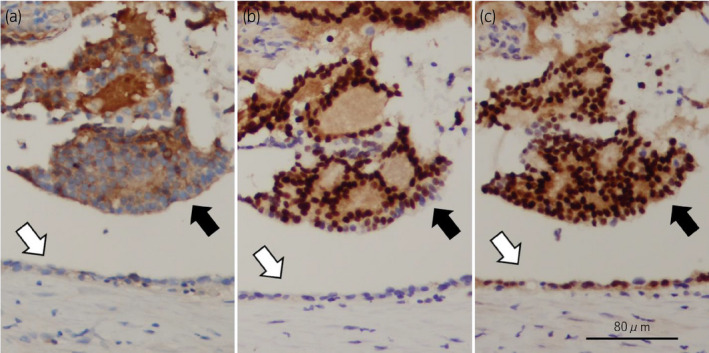
Immunopathological findings of the intracystic tissue (black arrows) and the cyst wall (white arrows). Immunohistochemistry for (a) PSA, (b) NKX 3.1, and (c) AR.

The metastatic lesion was a cystic mass (Fig. 2a), and the intracystic tissue was adenocarcinoma (Fig. 2b). Histopathological examination revealed no neuroendocrine features. The PSA level of intracystic fluid was 45 250 ng/mL. There was no lymphoid tissue in the metastatic lesion. The cyst wall was lined with ciliated cuboidal epithelium (Fig. 2c) containing lipofuscin in the cytoplasm (Fig. 2d). Immunohistochemistry revealed that intracystic tissue was diffusely positive for PSA, NKX 3.1 (NKX 3.1 expression is predominantly localized to prostate epithelium^4^), and AR, which was compatible with the metastasis of PCa. In contrast, the epithelium of the cyst wall was negative for PSA and NKX 3.1 and was positive for AR, which confirms that the cyst wall was not derived from the prostate but the seminal vesicle or vas deferens (Fig. 3). Thus pathological diagnosis was metastasis of PCa surrounded by the epithelium of the seminal vesicle or vas deferens.

The patient’s PSA level reached 35 ng/mL prior to surgery and decreased to 0.2 ng/mL 3 months post‐surgery. His PSA level remained at the nadir under normal serum testosterone level and no metastasis appeared for 3 years without additional treatment.

## Discussion

We observed a solitary metastasis of PCa which was surrounded by the epithelium of the seminal vesicle or vas deferens, and no recurrence appeared for 3 years after the surgical procedure.

Why was the metastatic lesion surrounded by the epithelium of the seminal vesicle or vas deferens? There is no definitive answer to this question. The metastatic lesion was apart from the seminal vesicle and was not connected to the vas deferens according to intraoperative findings. The lesion was not a metastasis to the normal seminal vesicle or vas deferens. We have three hypotheses. First, there was an ectopic seminal vesicle in the left external iliac area and a metastasis of PCa to the ectopic seminal vesicle occurred. There is a possibility that the ectopic seminal vesicle represents the persistence of embryonic remnants. To our knowledge, there is only one former report of an ectopic seminal vesicle, which was discovered in the rectal wall after an operation for rectal carcinoma.^5^ Second, the biopsy of the prostate for the diagnosis of PCa resulted in dissemination of PCa cells and epithelium of the seminal vesicle to the left external iliac obturator area. According to the initial magnetic resonance imaging, the PCa invaded into the left seminal vesicle, and thus the metastasis might have occured on the same side. However, there were no epithelium and stromal tissue of the seminal vesicles in the biopsy specimen of the prostate. The third hypothesis is that a metastasis of PCa to the left external iliac obturator area occurred first, and it induced differentiation to the tissue of the seminal vesicle around it. However, there was no lymphoid tissue in the metastatic lesion, so the site where the PCa first metastasized is unclear based on this hypothesis. Although the origin of the metastatic lesion is a matter of speculation, the first hypothesis seems to be acceptable.

The standard treatment option for PCa patients diagnosed with metastatic progression following curative radiation therapy has been ADT.^1^ This is considered a non‐curative treatment and markedly affects the patient’s quality of life.^6^ Increasing evidence indicates that MDT such as surgery or SBRT improves the prognosis of patients diagnosed with a limited number of PCa metastasis, so‐called oligorecurrent PCa. MDT is a promising approach for oligorecurrent PCa as it can help avoid or postpone ADT. A recent prospective, randomized, multicenter phase II trial of oligorecurrent PCa which compared surveillance or MDT concluded that ADT‐free survival was longer with MDT than with surveillance alone. This research suggested that MDT should be explored further in larger phase III trials.^7^ Triggiani *et al.* reported that metastasis‐directed SBRT prolonged systemic therapy‐free survival of patients with oligoprogressive CRPC in a retrospective multicenter study.^8^ MDT may be effective even for patients with oligoprogressive CRPC. In this case, the metastatic lesion grew larger despite ADT. Therefore, the patient was considered to develop CRPC. However, no recurrence appeared after the resection of the metastatic lesion, which suggested that metastasis was limited to one lesion and the surgical procedure was curative. MDT may possess a potential curative role and should be considered in certain subgroups of patients with metastatic PCa.

## Conflict of interest

The authors declare no conflict of interest.

## References

[1] van den Bergh RC , van Casteren NJ , van den Broeck T *et al* Role of hormonal treatment in prostate cancer patients with nonmetastatic disease recurrence after local curative treatment: a systematic review. Eur. Urol. 2016; 69: 802–20. 26691493 10.1016/j.eururo.2015.11.023

[2] Ost P , Bossi A , Decaestecker K *et al* Metastasis‐directed therapy of regional and distant recurrences after curative treatment of prostate cancer: a systematic review of the literature. Eur. Urol. 2015; 67: 852–63. 25240974 10.1016/j.eururo.2014.09.004

[3] Norihisa Y , Mizowaki T , Takayama K *et al* Detailed dosimetric evaluation of intensity‐modulated radiation therapy plans created for stage C prostate cancer based on a planning protocol. Int. J. Clin. Oncol. 2012; 17: 505–11. 21960356 10.1007/s10147-011-0324-1

[4] Abate‐Shen C , Shen MM , Gelmann E . Integrating differentiation and cancer: the Nkx3.1 homeobox gene in prostate organogenesis and carcinogenesis. Differentiation 2008; 76: 717–27. 18557759 10.1111/j.1432-0436.2008.00292.x PMC3683569

[5] Wader J , Kshirsagar A , Gajbi N , Kumbhar S . Ectopic prostatic and seminal vesicle tissue confusing as metastatic adenocarcinoma. Online J. Health Allied Sci. 2013; 12: 8.

[6] Studer UE , Whelan P , Wimpissinger F *et al* Differences in time to disease progression do not predict for cancer‐specific survival in patients receiving immediate or deffered androgen‐deprivation therapy for prostate cancer: final results of EORTC randomized trial 30891 with 12 years of follow‐up. Eur. Urol. 2014; 66: 829–38. 23932338 10.1016/j.eururo.2013.07.024

[7] Ost P , Reynders D , Decaestecker K *et al* Surveillance or metastasis‐directed therapy for oligometastatic prostate cancer recurrence: a prospective, randomized, multicenter phase II trial. J. Clin. Oncol. 2018; 36: 446–53. 29240541 10.1200/JCO.2017.75.4853

[8] Triggiani L , Mazzola R , Magrini SM *et al* Metastasis‐directed stereotactic radiotherapy for oligoprogressive castration‐resistant prostate cancer: a multicenter study. World J. Urol. 2019; 37: 2631–7. 30859273 10.1007/s00345-019-02717-7

